# Vitamin D levels and biomarkers of male fecundity: A study from the Danish National Birth Cohort

**DOI:** 10.1111/andr.70061

**Published:** 2025-05-18

**Authors:** Anne Gaml‐Sørensen, Nis Brix, Sandra Søgaard Tøttenborg, Christian Lindh, Karin Sørig Hougaard, Siri Eldevik Håberg, Gunnar Toft, Jens Peter Ellekilde Bonde, Cecilia Høst Ramlau‐Hansen

**Affiliations:** ^1^ Department of Public Health Aarhus University Aarhus Denmark; ^2^ Department of Clinical Genetics Aarhus University Hospital Aarhus Denmark; ^3^ Department of Occupational and Environmental Medicine Bispebjerg and Frederiksberg Hospital University of Copenhagen Copenhagen Denmark; ^4^ Department of Public Health University of Copenhagen København Denmark; ^5^ Division of Occupational and Environmental Medicine Lund University Lund Sweden; ^6^ National Research Centre for the Working Environment Copenhagen Denmark; ^7^ Centre for Fertility and Health Norwegian Institute of Public Health Oslo Norway; ^8^ Steno Diabetes Center Aarhus Aarhus University Hospital Aarhus Denmark

**Keywords:** hydroxyvitamin D_3_, male infertility, male reproductive health, reproductive hormones, semen quality, testes volume

## Abstract

**Background:**

Vitamin D is metabolised throughout the male reproductive system, suggesting a direct regulatory role of vitamin D in male reproduction.

**Objectives:**

To investigate the association between plasma vitamin D levels at sperm ejaculation and during spermatogenesis and biomarkers of male fecundity in young men.

**Materials and methods:**

From the Fetal Programming of Semen Quality cohort, Denmark, 2017–2019, 1047 young men provided a semen and a blood sample, and self‐measured their testes volume at a clinical visit. Plasma levels of vitamin D (25(OH)D_3_) and reproductive hormones were measured in the blood sample. Relative percentage differences in semen characteristics, testes volume and reproductive hormone levels were analysed according to measured vitamin D levels (categorised, continuous and as restricted cubic splines) at sperm ejaculation. Additionally, we used the seasonal variation in endogenous vitamin D synthesis to estimate individual vitamin D levels 3 months prior to sperm ejaculation (at initiation of spermatogenesis) in addition to 2 and 1 month before. This was analysed following the same strategy.

**Results:**

Compared to measured vitamin D levels >75 nmol/L, levels <25 nmol/L at sperm ejaculation were associated with lower total sperm count (‒15% [95% confidence interval: ‒33%; 8%]), and a higher proportion of non‐progressive and immotile spermatozoa (11% [95% confidence interval: 0%; 24%]). Lower measured vitamin D levels were also associated with higher oestradiol, lower sex hormone‐binding globulin and lower follicle‐stimulating hormone, in dose‐dependent manners. Vitamin D levels estimated before and during spermatogenesis yielded similar associations as vitamin D levels measured at sperm ejaculation.

**Discussion:**

By using the seasonal variation in endogen vitamin D synthesis, we were able to estimate individual vitamin D levels during spermatogenesis.

**Conclusion:**

Lower vitamin D levels before and during spermatogenesis and at sperm ejaculation were associated with lower total sperm count and sperm motility and an altered reproductive hormone profile.

## 1 INTRODUCTION

Vitamin D is an essential steroid hormone, classically known for its role in maintenance of calcium and phosphate homeostasis and bone health.^1^ However, evidence suggests that vitamin D may also be important for the reproductive system.^2^, ^3^, ^4^ Vitamin D metabolising enzymes—pivotal for activation of vitamin D—and the vitamin D receptor, through which activated vitamin D exerts its action, are widely distributed in the male reproductive system, including the hypothalamic‒pituitary‒gonadal axis,^5^, ^6^ the male reproductive tract and spermatozoa.^4^, ^7^, ^8^, ^9^ This suggests a direct regulatory role of vitamin D in the male reproductive system.

At present, three reviews^10^, ^11^, ^12^ and one meta‐analysis^13^ have evaluated the association between vitamin D levels and biomarkers of male fecundity. The association has been studied in five cross‐sectional studies in young men from the general population; however, current evidence is still inconclusive. Ramlau‐Hansen et al.,^14^ Józków et al.^15^ and Rudnicka et al.^16^ found no association between vitamin D levels and biomarkers of male fecundity in Danish, Polish and Spanish men. In another Danish study, Blomberg‐Jensen et al. found that men with vitamin D deficiency (<25 nmol/L) had a lower proportion of motile, progressive motile and morphologically normal spermatozoa compared with men with adequate vitamin D levels (>75 nmol/L).^17^ In American men, Hammoud et al. found that men with high vitamin D levels (>125 nmol/L) had lower sperm concentration and a lower proportion of motile, progressive motile and morphologically normal spermatozoa and that men with low vitamin D levels (<50 nmol/L) had lower total sperm count and a lower proportion of progressive motile spermatozoa compared with men with vitamin D levels of 50–125 nmol/L.^18^

Low vitamin D represents a potential modifiable factor for poor male fecundity that is of increasing concern in Western countries.^19^ We therefore aimed to investigate whether serum vitamin D levels were associated with semen characteristics, testes volume and reproductive hormones in the largest sample to date of young adult men from the general population. Additionally, vitamin D levels at the beginning (approximately 3 month prior to ejaculation of mature spermatozoa) or during the spermatogenetic cycle may constitute an important exposure window^20^; however, studies on vitamin D exposure during the early phases of the spermatogenic cycle is currently lacking. Using the seasonal variation in vitamin D levels because of variation in sun exposure that occurs at Northern latitudes during the year,^21^, ^22^ we additionally aimed to investigate whether vitamin D levels at initiation of spermatogenesis were associated with semen characteristics, testes volume and reproductive hormones.

## 2 MATERIALS AND METHODS

This study was conducted in the Fetal Programming of Semen Quality (FEPOS) cohort^23^ consisting of young men born 1998–2000 of women enrolled in the Danish National Birth Cohort (DNBC).^24^

In total, 5697 young men, whose mothers completed two gestational interviews and provided a gestational blood sample in the DNBC, were randomly and consecutively invited to participate between March 2017 and December 2019. The men lived in or near Copenhagen or Aarhus and were at least 18 years and 9 months old to be eligible for invitation. They were encouraged to decline participation if they had a vasectomy, received chemotherapy, or no or only one testis.^23^ In total, 1058 men (19%) filled in an online pre‐clinical survey and participated in a clinical visit, where they provided a semen sample, and 1047 further provided a blood sample in which vitamin D levels were analysed (Figure S1).

### 2.1 Vitamin D levels

Plasma 25‐hydroxyvitamin D_3_ (25(OH)D_3_) were measured in non‐fasting venous blood samples using 2D liquid chromatography–tandem mass spectrometry (LC–MS/MS; QTRAP 6500+; AB Sciex) at the Division of Occupational and Environmental Medicine, Lund University, according to Gaml‐Sorensen et al.^25^ No samples were below the limit of detection, which was 0.5 ng/mL. External and internal quality control were satisfactory (Supporting Information S1). The 25(OH)D_3_ levels were converted to nmol/L by multiplication with 2.496.

### 2.2 Male fecundity

Biomarkers of male fecundity included semen characteristics, testes volume and reproductive hormone levels. Participants attended one of two participating clinics at Department of Occupational and Environmental Medicine at Bispebjerg Hospital in Copenhagen and Department of Occupational Medicine at Aarhus University Hospital in Aarhus, respectively. Prior to semen sample collection at their home (18%) or at the clinic (82%), the participants were encouraged to stay abstinent for at least 2 days. Actual abstinence time was noted as was any potential spillage during collection and time of sampling. The participants self‐measured both testes using a Prader Orchidometer,^26^ and provided a blood sample at the clinical visit.

Biomarkers included the following semen characteristics: semen volume, sperm concentration, total sperm count, proportions of progressive motile spermatozoa and spermatozoa with normal morphology, DNA fragmentation index and high DNA stainability. The semen characteristics were manually assessed according to the World Health Organisation guidelines from 2010^27^ by one of two trained medical laboratory technicians. All methods were quality controlled throughout the data collection and all quality controls were satisfactory.^23^

The average volume of the two self‐measured testes was used as an outcome.

Levels of the following reproductive hormones were analysed in plasma from non‐fasting venous blood samples obtained at the clinical visit: testosterone, oestradiol, sex hormone‐binding globulin (SHBG), follicle‐stimulating hormone (FSH), luteinising hormone (LH) and free testosterone (calculated using the Vermeulen formula assuming a constant albumin concentration of 43 g/L).^28^ Testosterone and oestradiol were assessed using LC–MS/MS and SHBG, FSH and LH were assessed using immunoassays (Cobas 8000 e602; Roche Diagnostics).^23^

### 2.3 Covariates

Directed acyclic graphs were used to identify potential confounding variables (Figure S2).^29^ Information on the following covariates was included in all analyses: maternal pre‐pregnancy body mass index (BMI), maternal smoking and parental socioeconomic status (highest of the parents) in the first trimester, obtained from the first interview in the DNBC; maternal age at delivery obtained from the Medical Birth Register; the young men's smoking obtained from the pre‐clinical FEPOS survey; and their BMI and season of the clinical visit obtained from the clinical visit (Tables 1 and 2).

**TABLE 1:** Measured vitamin D: baseline characteristics and precision variables according to measured vitamin D levels at the clinical visit in 1047 young men from the Fetal Programming of Semen Quality (FEPOS) cohort, nested within the Danish National Birth Cohort, Denmark, 1998‒2019.

	Vitamin D levels				
	<25 nmol/L	25‒50 nmol/L	50‒75 nmol/L	>75 nmol/L	Missing (%)
*n* (%)	149 (14.2)	427 (40.8)	365 (34.9)	106 (10.1)	
Vitamin D level (nmol/L), median (range)^a^	19.1 (9.4‒24.9)	39.5 (25.2‒49.9)	60.7 (50.1‒74.8)	84.9 (75.7‒120)	
Maternal characteristics					
Parental socioeconomic status, *n* (%)					0
High‐grade professional	42 (28.2)	147 (34.4)	131 (35.9)	38 (35.8)	
Low‐grade professional	49 (32.9)	135 (31.6)	130 (35.6)	32 (30.2)	
Skilled or unskilled worker	52 (34.9)	122 (28.6)	89 (24.4)	31 (29.2)	
Student or economically inactive	6 (4.0)	23 (5.4)	15 (4.1)	5 (4.7)	
Maternal smoking in first trimester, *n* (%)					0
Non‐smoker	111 (74.5)	319 (74.7)	298 (81.6)	81 (76.4)	
0‒10 cigarettes/day	31 (20.8)	91 (21.3)	61 (16.7)	18 (17.0)	
>10 cigarettes/day	7 (4.7)	17 (4.0)	6 (1.6)	7 (6.6)	
Maternal pre‐pregnancy BMI (kg/m^2^), mean (SD)	23.5 (4.2)	22.6 (3.5)	22.9 (3.6)	22.3 (3.0)	≈2
Maternal age at delivery (years), mean (SD)	30.7 (4.5)	30.8 (3.9)	31.4 (4.2)	30.9 (4.2)	<1
Maternal vitamin D level (nmol/L), mean (SD)^b^	52 (22)	53 (22)	58 (22)	65 (24)	≈18
Men's characteristics					
Smoking status, *n* (%)					<1
No, never smoker	58 (38.9)	<218 (51.1)^c^	<188 (51.5)^c^	42 (39.6)	
No, former smoker	28 (18.8)	45 (10.6)	46 (12.7)	12 (11.3)	
Yes, occasional smoker	44 (29.5)	115 (27.0)	86 (23.7)	36 (34.0)	
Yes, every day smoker	19 (12.8)	49 (11.5)	45 (12.4)	16 (15.1)	
BMI, mean (SD)	22.6 (4.3)	22.6 (3.6)	22.3 (2.8)	22.7 (2.9)	<1
Comorbidities^d^					<1
Yes	<5 (<3.4)^c^	28 (6.6)	8 (2.2)	<5 (<4.7)^c^	
No	>144 (>96.6)^c^	398 (93.4)	355 (97.8)	>101 (>95.3)^c^	
Clinical characteristics					
Season at clinical visit, *n* (%)					0
Winter	52 (34.9)	84 (19.7)	53 (14.5)	7 (6.6)	
Spring	50 (33.6)	92 (21.5)	52 (14.2)	8 (7.5)	
Summer	7 (4.7)	75 (17.6)	123 (33.7)	49 (46.2)	
Fall	40 (26.8)	176 (41.2)	137 (37.5)	42 (39.6)	
Abstinence time, *n* (%)					<1
<2 days	53 (35.6)	168 (39.6)	106 (29.0)	34 (32.4)	
2‒3 days	57 (38.3)	<129 (30.2)^c^	119 (32.6)	<37 (34.9)^c^	
>3 days	39 (26.2)	130 (30.7)	140 (38.4)	35 (33.3)	
Spillage, *n* (%)					<1
No	<124 (83.2)^c^	352 (83.4)	<298 (81.6)^c^	88 (83.0)	
Yes	25 (16.9)	70 (16.6)	67 (18.5)	18 (17.0)	
Place of semen collection, *n* (%)					<1
At home	15 (10.1)	58 (13.7)	50 (13.8)	13 (12.3)	
At the clinic	<134 (89.9)^c^	364 (86.3)	<315 (86.3)^c^	93 (87.7)	
Time until motility analysis, *n* (%)					<1
≤60 min	<110 (73.8)^c^	313 (74.3)	<285 (78.1)^c^	76 (71.7)	
>60 min	39 (26.5)	108 (25.7)	80 (22.0)	30 (28.3)	
Time at blood sample collection, *n* (%)					0
Morning <12 pm	47 (31.5)	155 (36.3)	136 (37.3)	39 (36.8)	
Afternoon 12‒18 pm	82 (55.0)	229 (53.6)	193 (52.9)	55 (51.9)	
Evening >18 pm	20 (13.4)	43 (10.1)	36 (9.9)	12 (11.3)	

*Note*: Numbers in the table correspond to mean (SD), proportions (%) or p50 (range).

Abbreviations: BMI, body mass index; p50, 50th pseudo‐percentile; SD, standard deviation.

^a^ Reported as pseudo‐percentile and pseudo‐range calculated from the average of the five nearest values.

^b^ Only available in a subset of the mothers (*n* = 827).

^c^ Due to local data regulations (General Data Protection Regulation), it is not allowed to report numbers smaller than five, why the numbers in the table have been changed accordingly.

^d^ Any diabetes, hypo‐ or hyperthyroidism, or inflammatory bowel disease.

**TABLE 2:** Estimated vitamin D: baseline characteristics and precision variables according to estimated vitamin D levels at initiation of spermatogenesis in 1047 young men from the Fetal Programming of Semen Quality (FEPOS) cohort, nested within the Danish National Birth Cohort, Denmark, 1998‒2019.

	Vitamin D levels				
	<25 nmol/L	25‒50 nmol/L	50‒75 nmol/L	>75 nmol/L	Missing (%)
*n* (%)	155 (14.8)	407 (38.9)	370 (35.3)	115 (11.0)	
Vitamin D level (nmol/L), median (range)^a^	19.1 (8.2‒24.8)	38.8 (25.3‒49.9)	60.2 (50.1‒74.8)	84.2 (75.2‒121)	
Maternal characteristics					
Parental socioeconomic status, *n* (%)					0
High‐grade professional	47 (30.3)	140 (34.4)	131 (35.4)	40 (34.8)	
Low‐grade professional	50 (32.3)	131 (32.2)	124 (33.5)	>40 (34.8)^b^	
Skilled or unskilled worker	51 (32.9)	116 (28.5)	97 (26.2)	30 (26.1)	
Student or economically inactive	7 (4.5)	20 (4.9)	18 (4.9)	<5 (4.3)^b^	
Maternal smoking in first trimester, *n* (%)					0
Non‐smoker	115 (74.2)	302 (74.2)	303 (81.9)	89 (77.4)	
0‒10 cigarettes/day	34 (21.9)	88 (21.6)	60 (16.2)	19 (16.5)	
>10 cigarettes/day	6 (3.9)	17 (4.2)	7 (1.9)	7 (6.1)	
Maternal pre‐pregnancy BMI (kg/m^2^), mean (SD)	23.5 (4.0)	22.7 (3.7)	22.6 (3.5)	22.5 (2.9)	≈2
Maternal age at delivery (years), mean (SD)	30.7 (4.4)	30.9 (4.0)	31.2 (4.2)	31.2 (4.2)	<1
Maternal vitamin D level (nmol/L), mean (SD)^c^	51.9 (21.1)	53.4 (21.7)	57.6 (22.9)	62.9 (24.0)	≈18
Men's characteristics					
Smoking status, *n* (%)					<1
No, never smoker	65 (41.9)	<206 (50.6)^b^	<185 (50.0)^c^	50 (43.5)	
No, former smoker	18 (11.6)	50 (12.3)	45 (12.2)	16 (13.9)	
Yes, occasional smoker	42 (27.1)	107 (26.4)	95 (25.7)	37 (32.2)	
Yes, every day smoker	30 (19.4)	44 (10.9)	45 (12.2)	12 (10.4)	
BMI, mean (SD)	22.7 (4.3)	22.6 (3.6)	22.3 (2.8)	22.6 (2.9)	<1
Comorbidities^d^					<1
Yes	<5 (<3.2)^b^	25 (6.2)	10 (2.7)	<5 (<4.3)^b^	
No	>150 (>96.8)^b^	380 (93.8)	359 (97.3)	>110 (>95.7)^b^	
Clinical characteristics					
Season at clinical visit, *n* (%)					0
Winter	58 (37.4)	80 (19.7)	52 (14.1)	6 (5.2)	
Spring	46 (29.7)	87 (21.4)	59 (15.9)	10 (8.7)	
Summer	8 (5.2)	70 (17.2)	124 (33.5)	52 (45.2)	
Fall	43 (27.7)	170 (41.8)	135 (36.5)	47 (40.9)	
Abstinence time, *n* (%)					<1
<2 days	52 (33.5)	161 (39.9)	115 (31.1)	33 (28.9)	
2‒3 days	62 (40.0)	<126 (31.0)^b^	114 (30.8)	<40 (34.8)^b^	
>3 days	41 (26.5)	120 (29.7)	141 (38.1)	42 (36.8)	
Spillage, *n* (%)					<1
No	<128 (82.6)^b^	334 (83.1)	<305 (82.4)^b^	95 (82.6)	
Yes	27 (17.5)	68 (16.9)	65 (17.7)	20 (17.4)	
Place of semen collection, *n* (%)					<1
At home	20 (13.0)	50 (12.4)	50 (13.6)	16 (13.9)	
At the clinic	<135 (87.1)^b^	352 (87.6)	<320 (86.5)^b^	99 (86.1)	
Time until motility analysis, *n* (%)					<1
≤60 min	<113 (72.9)^b^	301 (75.1)	<287 (77.6)^b^	83 (72.2)	
>60 min	42 (27.5)	100 (24.9)	83 (22.6)	32 (27.8)	
Time at blood sample collection, *n* (%)					0
Morning <12 pm	49 (31.6)	157 (38.6)	129 (34.9)	42 (36.5)	
Afternoon 12‒18 pm	88 (56.8)	205 (50.4)	208 (56.2)	58 (50.4)	
Evening >18 pm	18 (11.6)	45 (11.1)	33 (8.9)	15 (13.0)	

*Note*: Numbers in the table correspond to mean (SD), proportions (%) or p50 (range).

Abbreviations: BMI, body mass index; p50, 50th pseudo‐percentile; SD, standard deviation.

^a^ Reported as pseudo‐percentile and pseudo‐range calculated from the average of the five nearest values.

^b^ Due to local data regulations (General Data Protection Regulation), it is not allowed to report numbers smaller than five, why the numbers in the table have been changed accordingly.

^c^ Only available in a subset of the mothers (*n* = 827).

^d^ Any diabetes, hypo‐ or hyperthyroidism, or inflammatory bowel disease.

### 2.4 Statistical analysis

Covariates and biomarkers of male fecundity were presented according to vitamin D levels (Tables 1, 2, S1 and S2) as proportions (%), means (standard deviation [SD]) and medians (interquartile range or range). All percentiles were reported as pseudo‐percentiles, which were calculated as the mean of the five values nearest to the actual percentile because of local regulations (General Data Protection Regulation, Regulation [EU], 2016/679 of 25 May 2018). Data management and statistical analyses were conducted in STATA 17.0 (StataCorp).

### 2.5 Measured vitamin D at sperm ejaculation

In the main analysis, we analysed measured vitamin D levels at sperm ejaculation. First, we analysed measured vitamin D in categories according to clinically relevant categorisations^3^, ^30^ as suggested previously^2^: <25 nmol/L (severe deficiency), 25–50 nmol/L (deficiency), 50–75 nmol/L (insufficiency) and >75 nmol/L (recommended). Then, continuously per SD (SD: 21 nmol/L) decrease to avoid introducing arbitrary cut‐offs and to examine potential dose dependency; and finally, as restricted cubic splines with three knots (at 10th percentile: 26 nmol/L, 50th percentile: 55 nmol/L, 90th percentile: 86 nmol/L).

In one subanalysis, we further adjusted for maternal vitamin D levels because it may be associated with both vitamin D levels and biomarkers of male fecundity in the adult sons.^25^ Maternal vitamin D levels were only available in a subset (*n* = 827) of the study population and was therefore only included in a subanalysis. Information on maternal vitamin D levels was obtained from first trimester maternal blood samples that was stored in the Danish National Biobank and analysed as descried above. In another subanalysis, we further adjusted the analysis for the presence of any comorbidities in the young men (any diabetes, hypo‐ or hyperthyroidism, or inflammatory bowel disease [yes, no]), obtained as self‐reported doctor‐diagnosed diseases from the pre‐clinical survey in FEPOS. Lastly, we collapsed the two groups with the highest measured vitamin D levels into one reference group (>50 nmol/L) because the threshold for a sufficient vitamin D level is still debated. For example, the Endocrine Society recommends maintaining a minimum of 75 nmol/L 25(OH)D, whereas the Institute of Medicine recommends maintaining a minimum of 50 nmol/L.^31^, ^32^, ^33^, ^34^

In sensitivity analyses, we assessed the robustness of the results regarding the limitations of the data collection. Although all semen characteristics were adjusted for place of semen sample collection (at home or in the clinic) and motility was further adjusted for time from ejaculation to analysis, we excluded participants collecting the semen sample at home in one sensitivity analysis. To acknowledge the recommendation that especially testosterone should be measured in the morning,^35^ we analysed the association between vitamin D and reproductive hormone levels only in participants that attended the clinic before 12 am in another sensitivity analysis.

### 2.6 Estimated vitamin D levels during spermatogenesis

Additionally, we examined the individual estimated level of vitamin D at initiation of spermatogenesis, that is, at 3 months prior to sperm ejaculation.^20^ In short, by regressing vitamin D levels on calendar month as an indicator variable, we predicted the population mean vitamin D level and estimated the relative difference based on this and the individual measured vitamin D. Then, we estimated vitamin D levels prior to sperm ejaculation by subtracting or adding the relative difference to the individually measured vitamin D levels (Supporting Information S2). Estimated vitamin D levels were also analysed in categories, continuously, and as restricted cubic spline plots as described above. In sensitivity analyses, we estimated vitamin D levels at different times during the spermatogenetic cycle (2 and 1 months prior to sperm ejaculation) to investigate additional potential important exposure windows of interest.

Semen characteristics and testes volume were analysed using a multivariable negative binomial regression model fitted by maximum likelihood estimation (STATA's ‐nbreg‐ package) according to vitamin D levels. Ratios were estimated with 95% confidence intervals (CIs) according to exposure groups, and relative percentage differences were then calculated: (ratio ‒ 1) × 100%. Reproductive hormone levels were log‐transformed and analysed according to vitamin D levels. Estimates were back transformed and presented as relative percentage differences with 95% CIs.

All models were adjusted for the potential confounding factors. In addition, we included variables expected to be strongly associated with the outcomes in the multivariable models to improve precision of the estimates. In models examining semen characteristics, we included abstinence time, place of semen sample collection (at home or in the clinic) and spillage of semen sample, although participants reporting spillage during sample collection (*n* = 180) were excluded from the models examining volume and total sperm count. Interval from ejaculation to analysis was further included in models examining motility. Although progressive motility was of main interest, we modelled non‐progressive + immotile in percentage in the statistical analyses to ensure optimal model fit. In models examining testes volume, abstinence time was further included. In models examining reproductive hormones, time of the day of blood sample drawing was included. Information on all precision variables was recorded at the clinical visit. All continuous variables were modelled as second‐order polynomials to allow for non‐linearity.

We fitted all models with selection weights using baseline parental characteristics and region of participant invitation to consider potential selection bias because of non‐participation^36^ as described in detail elsewhere.^37^ Robust standard errors were applied to account for the use of the weights and the clustering of siblings.

The negative binomial regression models were checked with Q–Q plots comparing the observed distributions against the model‐based distributions. Furthermore, standardised deviance residuals were plotted against model‐based predictions. The linear regression models were checked by plotting the model‐based residuals in Q–Q plots. Furthermore, the model‐based predictions were plotted against expected values. The model fits were acceptable.

## 3 RESULTS

Median age was 19 years and 2 months (range 18 years and 9 months to 21 years and 2 months). Median vitamin D level was 55 nmol/L (range 9.4‒120 nmol/L). In total, 149 men (14%) were severely vitamin D deficient (<25 nmol/L), 427 men (41%) were vitamin D deficient (25‒50 nmol/L), 365 men (35%) were vitamin D insufficient (50‒75 nmol/L) and 106 men (10%) had a recommended vitamin D level above 75 nmol/L at sperm ejaculation (Table 1).

Parents of men with measured severe vitamin D deficiency were less likely to be high‐grade professionals, and mothers had a higher BMI than mothers of men with higher vitamin D levels. Mothers of men with a recommended vitamin D level had higher vitamin D levels during pregnancy themselves. The men with the lowest vitamin D levels were more likely to smoke themselves (Table 1). Overall, the same patterns were observed for estimated vitamin D levels at initiation of spermatogenesis regarding the distribution of covariates (Table 2). The crude estimates of biomarkers of male fecundity according to measured and estimated vitamin D levels are presented in Tables S1 and S2.

### 3.1 Measured vitamin D at sperm ejaculation

Men with severe vitamin D deficiency at sperm ejaculation had lower total sperm count (‒15% [95% CI: ‒33%; 8%]), higher proportions of non‐progressive and immotile spermatozoa (11% [95% CI: 0%; 24%]), higher oestradiol (12% [95% CI: ‒6%; 34%]), higher free testosterone (7% [95% CI: ‒1%; 16%]), lower SHBG (‒15% [95% CI: ‒24%; ‒6%]), LH (‒6% [95% CI: ‒16%; 4%]) and FSH (‒11% [95% CI: ‒25%; 4%]), compared to men with recommended vitamin D levels.

We observed associations with a higher proportion of spermatozoa with normal morphology (7% [95% CI: 2%; 12%]), higher oestradiol (6% [95% CI: 1%; 10%]), lower SHBG (‒4% [95% CI: ‒7%; ‒2%]) and lower FSH (‒2% [95% CI: ‒6%; 2%]) per SD lower measured vitamin D levels (Table 3).

**TABLE 3:** Measured vitamin D: crude and adjusted^a^ (95% confidence intervals [CIs]) relative percentage differences in biomarkers of male fecundity according to measured vitamin D levels (nmol/L) at the clinical visit in categories and per standard deviation (SD)^b^ lower vitamin D in 1047 young men from the Fetal Programming of Semen Quality (FEPOS) cohort, nested within the Danish National Birth Cohort, Denmark, 1998‒2019.

		Main analysis		Subanalyses			Sensitivity analyses
	nmol/L	Crude	Adjusted (95% CI)^c^	Adjusted (95% CI)^d^	Adjusted (95% CI)^e^	Adjusted (95% CI)^f^	Adjusted (95% CI)^g^
Semen characteristics^h^							
Volume^i^	<25	‒9%	‒6% (‒18; 7)	‒9% (‒20; 4)	‒6% (‒18; 7)	‒6% (‒15; 5)	‒9% (‒22; 5)
	25‒50	‒7%	‒6% (‒15; 4)	‒9% (‒18; 1)	‒6% (‒15; 5)	‒5% (‒11; 2)	‒7% (‒17; 4)
	50‒75	0%	‒1% (‒11; 9)	‒3% (‒12; 8)	‒1% (‒11; 9)	Ref.	‒3% (‒13; 9)
	>75	Ref.	Ref.	Ref.	Ref.		Ref.
Per SD lower vitamin D		‒3%	‒1% (‒5; 2)	‒3% (‒6; 1)	‒1% (‒5; 2)	‒1% (‒5; 2)	‒2% (‒6; 1)
Concentration	<25	0%	‒4% (‒22; 18)	‒3% (‒22; 20)	‒5% (‒23; 17)	‒2% (‒17; 16)	1% (‒20; 26)
	25‒50	4%	6% (‒10; 24)	4% (‒12; 24)	6% (‒10; 24)	7% (‒4; 20)	12% (‒6; 34)
	50‒75	6%	2% (‒14; 20)	‒2% (‒18; 17)	1% (‒14; 20)	Ref.	7% (‒11; 29)
	>75	Ref.	Ref.	Ref.	Ref.		Ref.
Per SD lower vitamin D		0%	0% (‒5; 5)	1% (‒5; 6)	0% (‒6; 5)	0% (‒5; 5)	1% (‒5; 7)
Total sperm count^i^	<25	‒20%	‒15% (‒33; 8)	‒17% (‒35; 6)	‒16% (‒34; 7)	‒8% (‒24; 10)	‒13% (‒33; 13)
	25‒50	‒10%	‒8% (‒24; 11)	‒13% (‒30; 7)	‒7% (‒23; 12)	0% (‒12; 13)	‒3% (‒21; 20)
	50‒75	‒2%	‒9% (‒25; 11)	‒13% (‒30; 7)	‒9% (‒25; 10)	Ref.	‒5% (‒24; 18)
	>75	Ref.	Ref.	Ref.	Ref.		Ref.
Per SD lower vitamin D		‒5%	‒2% (‒8; 4)	‒3% (‒9; 4)	‒2% (‒8; 4)	‒2% (‒8; 4)	‒2% (‒8; 5)
Motility^j^	<25	3%	11% (0; 24)	7% (‒5; 20)	11% (0; 24)	8% (0; 18)	12% (0; 26)
	25‒50	‒7%	‒5% (‒13; 4)	‒7% (‒17; 3)	‒6% (‒14; 3)	‒5% (‒10; 1)	‒4% (‒12; 6)
	50‒75	‒5%	‒3% (‒11; 6)	‒2% (‒12; 8)	‒3% (‒11; 6)	Ref.	‒4% (‒12; 6)
	>75	Ref.	Ref.	Ref.	Ref.		Ref.
Per SD lower vitamin D		0%	1% (‒1; 4)	0% (‒3; 3)	1% (‒1; 4)	1% (‒1; 4)	2% (‒1; 5)
Morphology	<25	9%	4% (‒14; 27)	‒4% (‒21; 19)	4% (‒15; 26)	16% (1; 33)	5% (‒14; 28)
	25‒50	10%	7% (‒9; 26)	2% (‒15; 22)	7% (‒9; 26)	19% (8; 31)	12% (‒5; 32)
	50‒75	‒7%	‒11% (‒25; 5)	‒19% (‒33; ‒2)	‒11% (‒25; 5)	Ref.	‒6% (‒21; 12)
	>75	Ref.	Ref.	Ref.	Ref.		Ref.
Per SD lower vitamin D		7%	7% (2; 12)	5% (0; 11)	7% (2; 12)	7% (2; 12)	7% (1; 12)
DFI	<25	‒9%	‒6% (‒19; 9)	‒14% (‒26; 1)	‒6% (‒19; 9)	5% (6; 17)	‒2% (‒17; 15)
	25‒50	‒11%	‒10% (‒20; 3)	‒15% (‒26; ‒2)	‒10% (‒21; 2)	2% (‒5; 10)	‒6% (‒18; 8)
	50‒75	‒13%	‒17% (‒27; ‒6)	‒21% (‒32; ‒10)	‒17% (‒27; ‒6)	Ref.	‒16% (‒26; ‒3)
	>75	Ref.	Ref.	Ref.	Ref.		Ref.
Per SD lower vitamin D		‒1%	0% (‒4; 4)	‒1% (‒5; 3)	0% (‒4; 4)	0% (‒4; 4)	1% (‒10; 14)
HDS	<25	0%	0% (‒13; 14)	‒3% (‒15; 10)	0% (‒13; 14)	‒8% (‒17; 2)	3% (‒11; 18)
	25‒50	4%	5% (‒6; 17)	6% (‒5; 19)	4% (‒7; 16)	‒1% (‒8; 6)	6% (‒6; 19)
	50‒75	5%	5% (‒5; 17)	8% (‒3; 21)	5% (‒5; 17)	Ref.	4% (‒8; 14)
	>75	Ref.	Ref.	Ref.	Ref.		Ref.
Per SD lower vitamin D		‒1%	0% (‒4; 3)	‒2% (‒5; 2)	‒1% (‒4; 3)	0% (‒4; 3)	1% (‒3; 5)
Testes volume^k^							
Average testes volume	<25	‒4%	‒2% (‒10; 8)	1% (‒18; 12)	‒2% (‒11; 8)	‒5% (‒11; 2)	N/A
	25‒50	0%	2% (‒6; 10)	4% (‒5; 13)	1% (‒6; 10)	‒1% (‒6; 3)	N/A
	50‒75	3%	4% (‒4; 13)	8% (‒1; 17)	4% (‒4; 13)	Ref.	N/A
	>75	Ref.	Ref.	Ref.	Ref.		N/A
Per SD lower vitamin D		‒2%	‒1% (‒4; 1)	‒1% (‒3; 1)	‒2% (‒4; 1)	‒1% (‒4; 1)	N/A
Reproductive hormones^l^							
Testosterone	<25	‒3%	0% (‒8; 8)	‒3% (‒11; 6)	0% (‒8; 8)	‒3% (‒9; 3)	‒10% (‒20; 2)
	25‒50	0%	1% (‒5; 8)	‒1% (‒8; 7)	1%% (‒5; 8)	‒1% (‒5; 4)	‒7%% (‒16; 3)
	50‒75	2%	1% (‒5; 8)	1% (‒6; 9)	1% (‒5; 8)	Ref.	‒2% (‒12; 8)
	>75	Ref.	Ref.	Ref.	Ref.		Ref.
Per SD lower vitamin D		‒1%	0% (‒2; 2)	‒1% (‒3; 1)	0% (‒2; 2)	0% (‒2; 2)	‒3% (‒6; 0)
Oestradiol	<25	14%	12% (‒6; 34)	13% (‒6; 35)	13% (‒6; 35)	14% (1; 29)	‒2% (‒26; 31)
	25‒50	13%	10% (‒6; 27)	9% (‒8; 27)	9% (‒6; 27)	10% (1; 20)	‒5% (‒25; 20)
	50‒75	2%	0% (‒14; 17)	‒2% (‒17; 16)	0% (‒14; 17)	Ref.	‒10% (‒30; 15)
	>75	Ref.	Ref.	Ref.	Ref.		Ref.
Per SD lower vitamin D		7%	6% (1; 10)	6% (1; 10)	5% (1; 10)	6% (1; 10)	1% (‒5; 9)
SHBG	<25	‒14%	‒15% (‒24; ‒6)	‒14% (‒23; ‒4)	‒15% (‒24; ‒6)	‒12% (‒18; ‒5)	‒31% (‒43; ‒18)
	25‒50	‒8%	‒9% (‒17; 0)	‒8% (‒17; 1)	‒9% (‒17; 0)	‒6% (‒11; ‒1)	‒20% (‒31; ‒7)
	50‒75	‒2%	‒4% (‒12; 4)	‒4% (‒13; 5)	‒4% (‒12; 5)	Ref.	‒11% (‒22; 2)
	>75	Ref.	Ref.	Ref.	Ref.		Ref.
Per SD lower vitamin D		‒5%	‒4% (‒7; ‒2)	‒4% (‒6; ‒1)	‒4% (‒7; ‒2)	‒4% (‒7; ‒2)	‒9% (‒13; ‒5)
LH	<25	‒7%	‒6% (‒16; 4)	‒7% (‒17; 4)	‒7% (‒16; 4)	‒5% (‒12; 3)	‒7% (‒22; 11)
	25‒50	‒2%	‒1% (‒9; 7)	‒2% (‒11; 8)	‒2% (‒10; 7)	0% (‒6; 5)	0% (‒12; 14)
	50‒75	‒3%	‒2% (‒10; 7)	‒4% (‒13; 6)	‒2% (‒10; 7)	Ref.	3% (‒9; 17)
	>75	Ref.	Ref.	Ref.	Ref.		Ref.
Per SD lower vitamin D		‒1%	‒1% (‒3; 2)	‒1% (‒4; 2)	‒1% (‒4; 2)	‒1% (‒3; 2)	‒2% (‒6; 2)
FSH	<25	‒15%	‒11% (‒25; 4)	‒10% (‒24; 7)	‒11% (‒25; 4)	‒9% (‒18; 2)	‒20% (‒38; 3)
	25‒50	‒11%	‒8% (‒20; 5)	‒6% (‒19; 10)	‒8% (‒20; 5)	‒5% (‒12; 2)	‒19% (‒35; 2)
	50‒75	‒9%	‒6% (‒18; 8)	‒7% (‒21; 9)	‒5% (‒18; 9)	Ref.	‒5% (‒24; 18)
	>75	Ref.	Ref.	Ref.	Ref.		Ref.
Per SD lower vitamin D		‒3%	‒2% (‒6; 2)	‒2% (‒6; 3)	‒2% (‒6; 2)	‒2% (‒6; 2)	‒8% (‒14; ‒1)
Free testosterone	<25	6%	7% (‒1; 16)	6% (‒3; 15)	5% (‒4; 15)	3% (‒3; 9)	6% (‒7; 21)
	25‒50	7%	6% (‒1; 13)	5% (‒3; 13)	7% (0; 15)	2% (‒2; 7)	3% (‒6; 14)
	50‒75	6%	6% (‒2; 13)	5% (‒3; 13)	3% (‒1; 10)	Ref.	4% (‒5; 14)
	>75	Ref.	Ref.	Ref.	Ref.		Ref.
Per SD lower vitamin D		1%	1% (‒1; 3)	1% (‒1; 4)	2% (‒1; 4)	1% (‒1; 3)	2% (‒1; 5)

Abbreviations: DFI, DNA fragmentation index; FSH, follicle‐stimulating hormone; HDS, high DNA stainability; LH, luteinising hormone; SHBG, sex hormone‐binding globulin.

^a^ Adjusted for highest parental socioeconomic status, maternal smoking in pregnancy, maternal pre‐pregnancy body mass index (BMI), smoking, BMI and season.

^b^ SD = 21 nmol/L.

^c^ Main analysis.

^d^ Subanalysis further adjusted for maternal vitamin D levels in first trimester (available for *n* = 827).

^e^ Subanalysis further adjusted for comorbidities in the young men.

^f^ Subanalysis collapsing the two highest exposed groups into one reference group (>50 nmol/L).

^g^ Sensitivity analyses. Semen characteristics are analysed with restriction to the participants that collected the semen sample in the clinic (*n* > 906). Reproductive hormone levels are analysed with restriction to the participants that attended the clinic before 12 am (*n* = 377).

^h^ Further adjusted for abstinence time, spillage and place of semen sample collection.

^i^ Excluding samples with spillage (*n* = 180).

^j^ Further adjusted for interval between ejaculation and analysis. Due to model fit, the results are presented as the proportion of non‐progressive and immotile spermatozoa. Therefore, positive estimates should be interpreted as a decrease in progressive motility and vice versa.

^k^ Further adjusted for abstinence time.

^l^ Further adjusted for time of blood sample collection.

**FIGURE 1:**
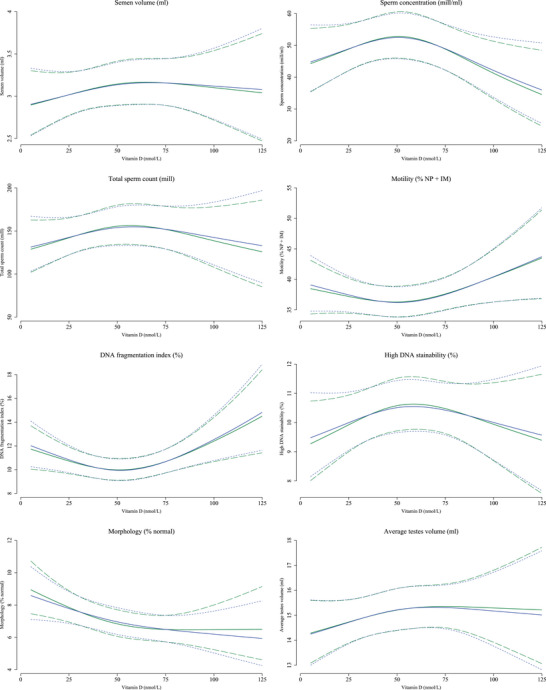
Restricted cubic spline plots of semen characteristics and testes volume according to measured vitamin D levels at sperm ejaculation (blue solid lines) with 95% confidence intervals (blue long‐dashed lines) and estimated vitamin D levels at initiation of spermatogenesis (green solid lines) with 95% confidence intervals (green short‐dashed lines). Semen characteristics and testes volume are presented for a reference man, born by parents where at least one was a high‐grade professional, whose mother was normal weight prior to the pregnancy, and was a non‐smoker in the first trimester of the pregnancy. The reference man was normal weight and was a non‐smoker. He attended the clinic during the fall season, had an abstinence time of 2.5 days, delivered his semen sample at the clinic, did not report any spillage of the semen sample and had his motility assessment performed 30 min after ejaculation. Knots are placed at the 10th percentile: 26 nmol/L, 50th percentile: 55 nmol/L and 90th percentile: 85 nmol/L for measured vitamin D levels at sperm ejaculation, and at the 10th percentile: 21 nmol/L, 50th percentile: 48 nmol/L and 90th percentile: 76 nmol/L for estimated vitamin D levels at initiation of spermatogenesis. IM, immotile motility; NP, non‐progressive motility.

**FIGURE 2:**
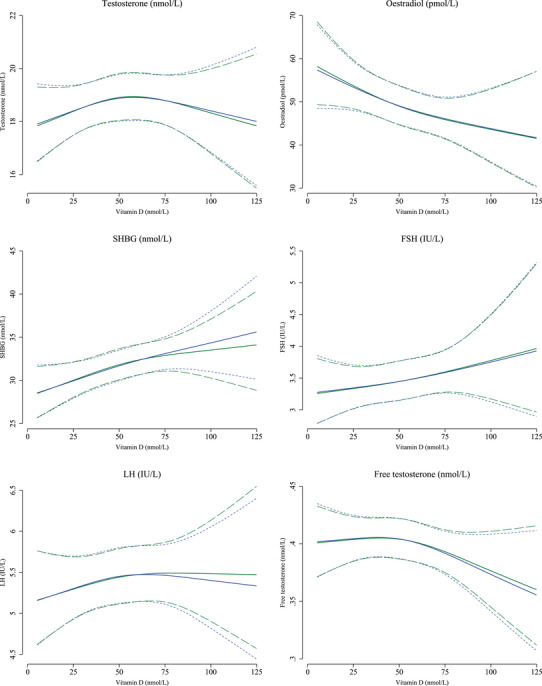
Restricted cubic spline plots of reproductive hormone levels according to measured vitamin D levels at sperm ejaculation (blue solid lines) with 95% confidence intervals (blue long‐dashed lines) and estimated vitamin D levels at initiation of spermatogenesis (green solid lines) with 95% confidence intervals (green short‐dashed lines). Reproductive hormone levels are presented for a reference man, born by parents where at least one was a high‐grade professional, whose mother was normal weight prior to the pregnancy, and was a non‐smoker in the first trimester of the pregnancy. The reference man was normal weight and was a non‐smoker. He attended the clinic during the fall season and had blood drawn for assessment of reproductive hormone levels from 12–18 pm. Knots are placed at the 10th percentile: 26 nmol/L, 50th percentile: 55 nmol/L and 90th percentile: 85 nmol/L for measured vitamin D levels at sperm ejaculation and at the 10th percentile: 21 nmol/L, 50th percentile: 48 nmol/L and 90th percentile: 76 nmol/L for estimated vitamin D levels at initiation of spermatogenesis. FSH, follicle‐stimulating hormone; LH, luteinising hormone; SHBG, sex hormone‐binding globulin.

In the adjusted spline plots, we saw indications that the semen characteristics, testes volume, testosterone and free testosterone were highest around 50 nmol/L vitamin D (Figures 1 and 2). For the remaining reproductive hormones, higher vitamin D levels were generally associated with lower oestradiol, higher SHBG, FSH and LH in a dose‐dependent manner (Figure 2).

Results were overall comparable across all subanalyses and in the sensitivity analysis of the semen characteristics excluding participants having collected the semen sample at home (Table 3). In the sensitivity analysis restricting the reproductive hormone analyses to participants that attended the clinic before 12 am, we found an association between severe vitamin D deficiency and lower testosterone (‒10% [95% CI: ‒20%; 2%]), and lower testosterone (‒3% [95% CI: ‒6%; 0%]) per SD lower measured vitamin D levels (Table 3).

### 3.2 Estimated vitamin D levels during spermatogenesis

When investigating estimated vitamin D levels at initiation of spermatogenesis, results were overall comparable with those from the main analyses (Table 4 and Figures 1 and 2). Associations between severe vitamin D deficiency and lower total sperm count were slightly stronger (‒20% [95% CI: ‒37%; 0%]) 1‒2 months before ejaculation (Table 4).

**TABLE 4:** Estimated vitamin D: crude and adjusted^a^ (95% confidence intervals [CIs]) relative percentage differences in biomarkers of male fecundity according to estimated vitamin D levels (nmol/L) during spermatogenesis in categories and per standard deviation (SD)^b^ lower vitamin D in 1047 young men, the Fetal Programming of Semen Quality (FEPOS) Cohort, Denmark, 2017‒2019.

		Main analysis		Sensitivity analyses	
			Adjusted (95% CI)^c^	Adjusted (95% CI)^d^	Adjusted (95% CI)^e^
	nmol/L	Crude	3 months prior to sperm ejaculation	2 months prior to sperm ejaculation	1 month prior to sperm ejaculation
Semen characteristics^f^					
Volume^g^	<25	‒7%	‒7% (‒19; 6)	‒8% (‒20; 5)	‒9% (‒20; 4)
	25‒50	‒2%	‒7% (‒16; 3)	‒6% (‒15; 4)	‒7% (‒16; 3)
	50‒75	0%	‒2% (‒12; 8)	‒3% (‒12; 8)	‒2% (‒12; 8)
	>75	Ref.	Ref.	Ref.	Ref.
Per SD lower vitamin D		‒3%	‒2% (‒5; 2)	‒1% (‒5; 2)	‒2% (‒5; 2)
Concentration	<25	‒3%	‒8% (‒25; 13)	‒9% (‒26; 13)	‒6% (‒24; 16)
	25‒50	2%	4% (‒12; 22)	5% (‒11; 24)	4% (‒11; 23)
	50‒75	2%	0% (‒15; 18)	2% (‒14; 20)	‒1% (‒16; 17)
	>75	Ref.	Ref.	Ref.	Ref.
Per SD lower vitamin D		0%	0% (‒5; 5)	0% (‒6; 5)	0% (‒6; 5)
Total sperm count^g^	<25	‒18%	‒17% (‒34; 6)	‒20% (‒37; 0)	‒20% (‒37; 0)
	25‒50	‒5%	‒11% (‒26; 8)	‒9% (‒25; 10)	‒10% (‒26; 9)
	50‒75	‒1%	‒11% (‒27; 8)	‒11% (‒27; 8)	‒11% (‒27; 8)
	>75	Ref.	Ref.	Ref.	Ref.
Per SD lower vitamin D		‒5%	‒2% (‒8; 4)	‒2% (‒8; 4)	‒2% (‒9; 3)
Motility^h^	<25	0%	13% (1; 26)	11% (‒1; 24)	12% (0; 25)
	25‒50	‒6%	‒3% (‒11; 5)	‒4% (‒11; 5)	‒4% (‒12; 5)
	50‒75	‒6%	‒1% (‒9; 8)	‒3% (‒11; 6)	‒2% (‒10; 7)
	>75	Ref.	Ref.	Ref.	Ref.
Per SD lower vitamin D		0%	2% (‒1; 4)	1% (‒1; 4)	2% (‒1; 4)
Morphology	<25	12%	5% (‒13; 28)	8% (‒11; 31)	7% (‒12; 30)
	25‒50	10%	7% (‒9; 26)	9% (‒8; 29)	6% (‒10; 26)
	50‒75	‒8%	‒9% (‒23; 8)	‒7% (‒22; 10)	‒10% (‒24; 6)
	>75	Ref.	Ref.	Ref.	Ref.
Per SD lower vitamin D		7%	7% (2; 12)	7% (2; 12)	7% (2; 12)
DFI	<25	‒9%	‒7% (‒20; 8)	‒7% (‒20; 8)	‒7% (‒20; 7)
	25‒50	‒11%	‒9% (‒20; 3)	‒8% (‒20; 4)	‒10% (‒20; 3)
	50‒75	‒13%	‒16% (‒26; ‒5)	‒15% (‒25; ‒4)	‒17% (‒27; ‒6)
	>75	Ref.	Ref.	Ref.	Ref.
Per SD lower vitamin D		‒1%	0% (‒4; 4)	0% (‒4; 4)	0% (‒4; 4)
HDS	<25	‒2%	0% (‒12; 13)	‒1% (‒14; 13)	‒2% (‒14; 11)
	25‒50	‒1%	7% (‒4; 19)	2% (‒9; 14)	6% (‒5; 18)
	50‒75	4%	8% (‒3; 19)	4% (‒7; 16)	5% (‒5; 17)
	>75	Ref.	Ref.	Ref.	Ref.
Per SD lower vitamin D		‒1%	‒1% (‒4; 3)	‒1% (‒4; 3)	0% (‒4; 3)
Testes volume^i^					
Average testes volume	<25	‒6%	‒3% (‒11; 7)	‒2% (‒11; 7)	‒2% (‒10; 8)
	25‒50	‒2%	2% (‒5; 10)	2% (‒5; 10)	2% (‒5; 10)
	50‒75	‒1%	4% (‒4; 13)	4% (‒4; 13)	4% (‒4; 13)
	>75	Ref.	Ref.	Ref.	Ref.
Per SD lower vitamin D		‒2%	‒1% (‒4; 1)	‒1% (‒4; 1)	‒1% (‒4; 1)
Reproductive hormones^j^					
Testosterone	<25	‒2%	‒2% (‒9; 6)	‒2% (‒9; 6)	‒2% (‒9; 6)
	25‒50	3%	0% (‒6; 7)	0% (‒6; 7)	0% (‒7; 6)
	50‒75	3%	2% (‒5; 9)	2% (‒5; 9)	1% (‒5; 9)
	>75	Ref.	Ref.	Ref.	Ref.
Per SD lower vitamin D		‒1%	‒1% (‒3; 1)	‒1% (‒3; 1)	‒1% (‒3; 1)
Oestradiol	<25	21%	20% (1; 43)	22% (3; 44)	20% (1; 42)
	25‒50	16%	15% (‒1; 33)	15% (‒1; 33)	13% (‒3; 30)
	50‒75	4%	7% (‒9; 25)	7% (‒8; 25)	5% (‒10; 22)
	>75	Ref.	Ref.	Ref.	Ref.
Per SD lower vitamin D		7%	6% (2; 11)	7% (2; 11)	6% (2; 11)
SHBG	<25	13%	‒13% (‒22; ‒4)	‒14% (‒21; ‒5)	‒14% (‒20; ‒4)
	25‒50	‒4%	‒8% (‒16; 0)	‒8% (‒16; 0)	‒9% (‒16; 0)
	50‒75	‒3%	‒3% (‒12; 5)	‒3% (‒11; 6)	‒4% (‒12; 5)
	>75	Ref.	Ref.	Ref.	Ref.
Per SD lower vitamin D		‒1%	‒4% (‒6; ‒2)	‒4% (‒6; ‒2)	‒4% (‒6; ‒2)
LH	<25	‒3%	‒7% (‒16; 3)	‒7% (‒16; 3)	‒7% (‒16; 3)
	25‒50	‒1%	‒2% (‒10; 6)	‒2% (‒9; 7)	‒2% (‒10; 6)
	50‒75	0%	‒3% (‒11; 5)	‒3% (‒11; 5)	‒3% (‒11; 5)
	>75	Ref.	Ref.	Ref.	Ref.
Per SD lower vitamin D		‒1%	‒1% (‒4; 1)	‒1% (‒4; 1)	‒1% (‒4; 1)
FSH	<25	‒13%	‒12% (‒24; 2)	‒14% (‒26; 0)	‒14% (‒26; 0)
	25‒50	‒9%	‒10% (‒21; 3)	‒10% (‒20; 3)	‒9% (‒20; 4)
	50‒75	‒8%	‒6% (‒18; 8)	‒6% (‒19; 7)	‒5% (‒17; 9)
	>75	Ref.	Ref.	Ref.	Ref.
Per SD lower vitamin D		‒4%	‒3% (‒7; 1)	‒3% (‒7; 0)	‒3% (‒7; 1)
Free testosterone	<25	5%	16% (‒2; 15)	6% (‒2; 15)	7% (‒2; 15)
	25‒50	6%	5% (‒2; 13)	6% (‒1; 13)	5% (‒2; 12)
	50‒75	4%	4% (‒3; 12)	5% (‒2; 13)	5% (‒3; 12)
	>75	Ref.	Ref.	Ref.	Ref.
Per SD lower vitamin D		2%	2% (0; 4)	2% (0; 4)	2% (0; 4)

Abbreviations: DFI, DNA fragmentation index; FSH, follicle‐stimulating hormone; HDS, high DNA stainability; LH, luteinising hormone; SHBG, sex hormone‐binding globulin.

^a^ Adjusted for highest parental socioeconomic status, maternal smoking in pregnancy, maternal pre‐pregnancy body mass index (BMI), smoking, BMI and season.

^b^ SD = 21 nmol/L.

^c^ Main analysis with estimated vitamin D levels 3 month prior to sperm ejaculation.

^d^ Subanalysis with estimated vitamin D levels 2 months prior to sperm ejaculation.

^e^ Subanalysis with estimated vitamin D levels 1 month prior to sperm ejaculation.

^f^ Further adjusted for abstinence time, spillage and place of semen sample collection.

^g^ Excluding samples with spillage (*n* = 180).

^h^ Further adjusted for interval between ejaculation and analysis. Due to model fit, the results are presented as the proportion of non‐progressive and immotile spermatozoa. Therefore, positive estimates should be interpreted as a decrease in progressive motility and vice versa.

^i^ Further adjusted for abstinence time.

^j^ Further adjusted for time of blood sample collection.

## 4 DISCUSSION

### 4.1 Key results

In this large cohort of young men from a population‐based cohort, we found that men with severe vitamin D deficiency had lower total sperm count and a higher proportion of non‐progressive and immotile spermatozoa, which translates into a lower proportion of progressive motile spermatozoa compared with men with recommended vitamin D levels. They additionally had lower LH and FSH. Moreover, low vitamin D levels were associated with an altered reproductive hormone profile characterised by higher oestradiol and lower SHBG. Surprisingly, men with severe vitamin D deficiency also had a higher proportion of morphologically normal spermatozoa compared with men with higher vitamin D levels. This could represent a chance finding because it has no obvious biological explanation. Results were overall similar for measured vitamin D levels at sperm ejaculation and estimated vitamin D levels during spermatogenesis.

### 4.2 Strengths and limitations

We analysed the association between vitamin D levels and biomarkers of male fecundity in the large FEPOS cohort, nested within the population‐based DNBC. Both vitamin D levels and biomarkers of male fecundity were measured using state‐of‐the‐art methods that were continuously quality controlled. Additionally, we took prenatal risk factors for poor male fecundity into account and applied inverse probability of selection weights in all analyses.

We consider the risk of selection bias limited, although the participation rate was low (19%). We have previously shown that potential selection bias because of non‐participation in the FEPOS cohort is unlikely to affect measures of associations, when investigating risk factors for poor male fecundity in this cohort.^38^ Additionally, participation is unlikely to be associated with biomarkers of male fecundity because the men were young and most likely unaware of their fecundity. Lastly, we considered potential selection bias because of possible differential loss‐to‐follow‐up using selection weights using the comprehensive baseline information.

We used a quality‐controlled objective measure of vitamin D, that is, plasma 25(OH)D_3_. This is a major study strength. By measuring plasma 25(OH)D_3_, we considered the contributions from both sunlight and intake of vitamin D from dietary sources, including supplements. The plasma half‐life of 25(OH)D_3_ is approximately 2–3 weeks^30^ and is an established biomarker in determining long‐term exposure.^31^ Potential measurement errors in vitamin D levels are expected to be minor, independent and non‐differential regarding biomarkers of male fecundity.

Likewise, we expect that any potential measurement errors in the biomarkers of male fecundity were most likely non‐differential regarding vitamin D levels. The laboratory technicians analysing the biomarkers of male fecundity were blinded to the participants’ exposure status. The laboratory measurements of the semen characteristics were quality‐controlled throughout the data collection and met given standards for semen analyses.^23^ The within individual variation in the semen characteristics may not introduce any systematic errors^39^; however, the large variation in the data may result in type II errors and, hence, limit our ability to detect true associations, if any. Testes volume may be underestimated for some participants^26^; however, this is likely non‐differential according to vitamin D levels. We accounted for the circadian fluctuations in reproductive hormone levels by adjusting for time of day of blood sampling. However, the sensitivity analysis restricting the reproductive hormone analyses to participants that attended the clinic before 12 am revealed that we might have been unable to detect an association between lower vitamin D levels and lower testosterone because of the inclusion of participants throughout the day.

Although we cannot exclude the risk of bias because of unmeasured confounding, we included several potential confounding factors from prenatal life, thus overcoming a major limitation compared with most previous research within this research area.

### 4.3 Interpretation

Research has found no association between vitamin D levels and biomarkers of male fecundity.^10^, ^11^, ^12^, ^13^ However, consistent with our findings, some studies have found an association between lower vitamin D levels, and lower total sperm count^10^, ^11^, ^12^, ^13^, ^18^, ^40^ and a lower proportion of motile spermatozoa,^10^, ^11^, ^12^, ^13^, ^17^ both in men from the general population and in selected samples of infertile men.^40^, ^41^ Given the high proportion of men with low vitamin D levels as seen in our study, this points towards a potential area for prevention of low male fecundity.

Most of the previous studies presented results adjusted for season as a potential confounder. The association between season and vitamin D in relation to male fecundity is complex. On one hand, a high vitamin D level may be beneficial for biomarkers of male fecundity, for example, some studies have suggested that higher vitamin D levels were associated with a higher proportion of motile spermatozoa and higher total sperm count,^10^, ^11^, ^12^, ^13^, ^17^ as also backed up in our study. On the other hand, many studies suggest that biomarkers of male fecundity, including total sperm count and the proportion of progressive motile spermatozoa, were lower during the summer than during the winter and vice versa.^42^, ^43^, ^44^, ^45^, ^46^ Simultaneously, vitamin D levels are highest during the summer and lowest during the winter because of the endogenous synthesis in skin following exposure to sunlight.^21^, ^22^ These contrasting associations highlights the necessity to carefully consider the potential for confounding by season.

We too adjusted for season in all analyses. In addition, we exploited the strong association between season and vitamin D by estimating vitamin D levels at initiation of spermatogenesis. By doing do, we were able to estimate the vitamin D level from the initiation of spermatogenesis, during spermatogenesis up until the time of sperm ejaculation. Our results were similar across all analyses. Therefore, we could not determine, which time point during spermatogenesis is the most important for male fecundity to maintain a sufficient vitamin D level, only that this is of importance.

At the time of data collection, there were no official recommendations regarding vitamin D intake in Denmark; however, from 2020, all healthy adults are advised to take 10 µg of vitamin D supplementation daily during the winter half‐year. Moreover, the Danish Health authorities recommend maintaining a minimum plasma vitamin D level of 50 nmol/L.^47^ Due to differences in recommendations,^31^, ^32^, ^33^, ^34^ we collapsed the two groups with highest vitamin D levels into one reference group (>50 nmol/L) in a subanalysis. This did not change the results.

Endogenous vitamin D synthesis occurs only during summer at northern latitudes and food fortification with vitamin D is not employed in Denmark. As a result, sunlight remains by far the main contributor to vitamin D levels in this study. Since potential food fortification with vitamin D and seasonal changes in environmental exposures might modify the association between vitamin D levels and biomarkers of male fecundity, our results may primarily be generalisable to similar populations.

In conclusion, we found that lower vitamin D levels were associated with lower total sperm count and proportion of progressive spermatozoa and an altered reproductive hormone profile, characterised by higher oestradiol and lower SHBG. The associations were of a similar magnitude for vitamin D levels estimated at initiation of spermatogenesis and vitamin D levels measured at the time of sperm ejaculation. Although this suggests that no exposure windows are of particular interest regarding the potential regulation of the male reproductive system by vitamin D, future follow‐up studies with repeated measurements of both vitamin D levels and biomarkers of fecundity could, however, add to the existing literature.

## AUTHOR CONTRIBUTIONS

The funding for FEPOS was acquired by Sandra Søgaard Tøttenborg and Jens Peter Ellekilde Bonde. Cecilia Høst Ramlau‐Hansen acquired the funding for this specific study, and Christian Lindh acquired the data on vitamin D levels. The data collection in FEPOS was planned and headed by Gunnar Toft, Sandra Søgaard Tøttenborg, Karin Sørig Hougaard, Jens Peter Ellekilde Bonde and Cecilia Høst Ramlau‐Hansen. Anne Gaml‐Sørensen planned this study with Cecilia Høst Ramlau‐Hansen. The analytic strategy was designed by Anne Gaml‐Sørensen, Nis Brix, Jens Peter Ellekilde Bonde and Cecilia Høst Ramlau‐Hansen. Anne Gaml‐Sørensen performed the statistical analyses and wrote the first draft. All authors had full access to all the data in the study, all authors interpreted the data, revised the manuscript critically, approved and accepted responsibility of the final manuscript.

## CONFLICT OF INTEREST STATEMENT

The authors declare they have no conflicts of interest.

## Supporting information



Supporting Information

Supporting Information

## Data Availability

The dataset analysed in the study is not publicly available because of national data security legislation on sensitive personal data. Researchers may apply for access to data from the DNBC. Please see https://www.dnbc.dk/data‐available or write to dnbc‐research@ssi.dk for additionally information.
