# Supplementary material for: Vitamin D levels and biomarkers of male fecundity: A study from the Danish National Birth Cohort
Source: Andrology. 2025 May 18;14(3):847–62. doi: 10.1111/andr.70061 (PMC12917574; doi:10.1111/andr.70061)
Supplement: Supplementary file 1 — Supporting Information [file ANDR-14-847-s001.docx]

**Supplementary material**

**Article title:** Vitamin D levels and biomarkers of male fecundity: a study from The Danish National Birth Cohort

**Authors:** Anne Gaml-Sørensen, Nis Brix, Sandra Søgaard Tøttenborg, Christian Lindh, Karin Sørig Hougaard, Siri Eldevik Håberg, Gunnar Toft, Jens Peter Ellekilde Bonde, Cecilia Høst Ramlau-Hansen

**Journal:** Andrology

**Correspondence:** Anne Gaml-Sørensen, Bartholins Allé 2, Aarhus University, 8000 Aarhus C, Denmark. Tel: +45 40868183; E-mail: ags@ph.au.dk ; ORCiD iD: 0000-0002-5242-939X

**Page 2:** Supplementary Figure 1: Flowchart

**Page 3:** Supplementary text 1: Analysis of 25(OH)D_3_

**Page 4:** Supplementary Figure 2: Directed acyclic graph (DAG)

**Page 5:** Supplementary text 2: Estimating vitamin D

**Page 7:** Supplementary Figure 3: Vitamin D levels according to date at sperm ejaculation

**Page 8:** Supplementary Figure 4: Vitamin D levels according to month at sperm ejaculation

**Page 9:** Supplementary Table S1. Crude biomarkers of male fecundity according to measured vitamin D.

**Page 10:** Supplementary Table S2. Crude biomarkers of male fecundity according to estimated vitamin D.

**Supplementary Figure 1: Flowchart**

**Flowchart.** Flowchart of the inclusion into the study on vitamin D levels and biomarkers of male fecundity. Of the 5697 young men invited to the fetal programming og semen quality (FEPOS) cohort, 1047, corresponding to 18%, were included in the study. The FEPOS Cohort, 2017 – 2019, Denmark.

| n = 5697 invited to FEPOS | | | | |  |  |  |  |
| --- | --- | --- | --- | --- | --- | --- | --- | --- |
|  |  |  |  |  |  | Young men not responding to FEPOS questionnaire (n = 4524) | | |
|  |  |  |  |  |  |  |  |  |
| n = 1173 answered the FEPOS questionnaire | | | | |  |  |  |  |
|  |  |  |  |  |  | Young men not participating in clinical examination (n = 115) | | |
|  |  |  |  |  |  |  |  |  |
| n = 1058 participated in the clinical examination | | | | |  |  |  |  |
|  |  |  |  |  |  | Young men not having a blood sample sufficient for vitamin D analysis (n = 11) | | |
|  |  |  |  |  |  |  |  |  |
| n = 1047 included in the analysis | | | | |  |  |  |  |
|  |  |  |  |  |  |  |  |  |

**Supplementary text 1: Analysis of 25(OH)D_3_**

A full method description is available in Gaml-Sorensen et al. 2023.^1^ Briefly, aliquots of 100 µL of plasma sample were added with isotopically labelled internal standards precipitated with acetonitril. Quantitative analysis was conducted using a triple quadrupole linear ion trap mass spectrometer (MS) equipped with TurboIonSpray sources (QTRAP6500+, AB Sciex, Framingham, MA, USA) coupled to a liquid chromatography system (UFLCXR, Shimadzu Corporation, Kyoto, Japan; LC/MS/MS). Separation was performed using a two-dimensional system with Reprosil Gold (C4; 3µm 20x4.6mm, Dr Maisch) and Raptor Biphenyl (2.7-µm 100 x 4.6 mm; Restek).

Four chemical blanks samples, calibration standards, two reference samples prepared in-house (QC1, QC2) and two QC samples from Chromsystems Instruments & Chemicals GmbH (MassCheck; Gräfelfing, Germany) were analysed as internal and external control for all analytical batches. The samples were analysed in random order.

The two in-house prepared reference samples had a mean level of QC1 31 ng/mL with a coefficient of variation (CV) of 8%. QC2 had a mean level of 34 ng/mL and a CV of 7%. For the two QC samples from Chromsystems Instruments & Chemicals GmbH (MassCheck; Gräfelfing, Germany), the target value for QC Level I was 16.7 ng/mL (range 13.3–20.0 ng/mL) and for QC Level II was 37.7 ng/mL (range 30.2–45.3 ng/mL). The level quantified in our analysis was 14.5 ng/mL (CV 10%) for Level I and 33.5 ng/mL (CV 8%) for Level II.

*References*

1. Gaml-Sorensen A, Brix N, Haervig KK, et al. Maternal vitamin D levels and male reproductive health: a population-based follow-up study. Eur J Epidemiol. May 2023;38(5):469-484. https://doi.org10.1007/s10654-023-00987-5.

**Supplementary Figure 2: Directed acyclic graph (DAG)**

**Directed acyclic graph (DAG).** DAG illustrating the causal framework underlying the study on vitamin D levels and markers of male fecundity. Boxes indicate conditioning in the statistical analyses. The FEPOS Cohort, 1998 – 2019, Denmark.

Abbreviations: BMI: body mass index


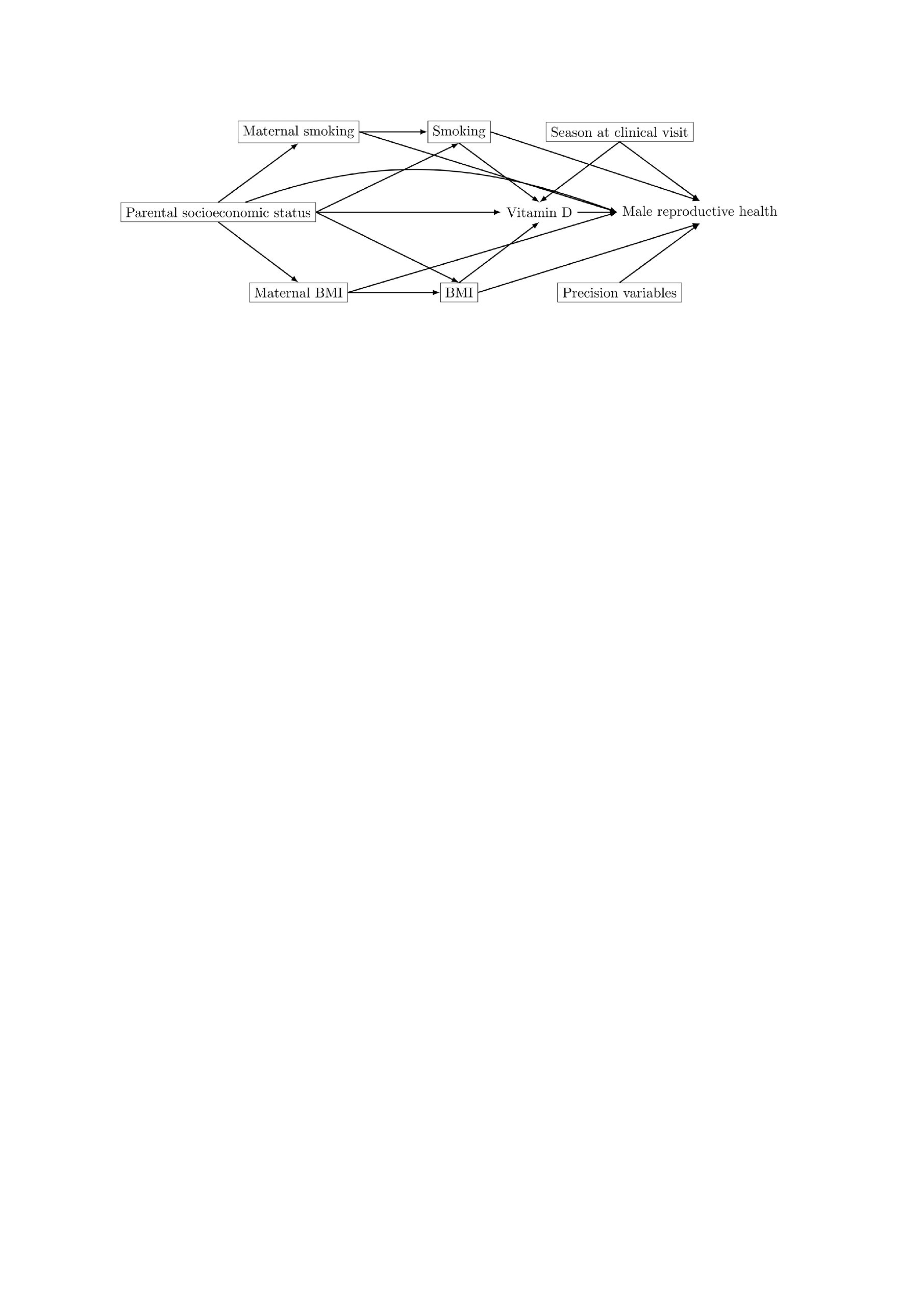


**Supplementary text 2: Estimating vitamin D**

We aimed to estimate vitamin D levels at the beginning of the spermatogenesis and during the spermatogenetic cycle. This was done by taking advantage of the seasonality in sun exposure and in the endogenous synthesis of vitamin D in the human skin that occurs at Northern latitudes during the summer.^1-3^ We used season to estimate vitamin D levels, since the principal source of vitamin D comes from synthetization of vitamin D_3_ from 7-dehydrocolesterol in the human skin following exposure to sunlight.^1,4^

Vitamin D levels fluctuated throughout the data collection from May 8^th^, 2017, until December 19^th^, 2019 (Supplementary Figure 3). We used calendar month to predict mean vitamin D for the population and the individual relative deviations from the predicted means. This was done in an ordinary least square regression model fitted by regressing vitamin D levels on calendar month as an indicator variable. Vitamin D levels according to calendar month is presented in Supplementary Figure 4. Post estimation, we predicted the residuals (i.e., the individual deviation from the predicted mean vitamin D levels according to calendar month), and based on this, we obtained the relative difference from the population average.

Since the spermatogenesis is expected to take place through approximately 74 days,^5^ three, two, and one month prior to ejaculation (i.e., prior to attending the clinical visit) was deemed appropriate time points for vitamin D estimation. Hence, the relative difference from the population average was subtracted from or added to the predicted mean three, two and one month prior to sperm ejaculation (i.e., the individual vitamin D level during spermatogenesis was estimated as the predicted vitamin D level based on calendar month three, two and one month prior to attending the clinical visit + individual relative difference from the population average at the time of attending the clinical visit).

For example, a participant attends the clinic in the beginning of June. Predicted mean vitamin D in June is around 50 nmol/L vitamin D. This participant has, however, a measured vitamin D level of 80 nmol/l. This is a relative deviation from the population average of 60% (30/50 = 0.6). Three months prior to attending the clinic, i.e., in March, the mean predicted vitamin D was much lower at around 35 nmol/l. The participant’s vitamin D at this time would then be estimated to be 60% higher than the predicted vitamin D level in March, i.e., the estimated vitamin D level for this participant would then be 35 nmol/l + 35 nmol/l x 0,6 = 56 nmol/l.

*References*

1. Holick MF. Ultraviolet B Radiation: The Vitamin D Connection. In: Ahmad SI, ed. *Ultraviolet Light in Human Health, Diseases and Environment*. Cham: Springer International Publishing; 2017:137-154.

2. Hansen L, Tjonneland A, Koster B, et al. Vitamin D Status and Seasonal Variation among Danish Children and Adults: A Descriptive Study. *Nutrients*. Nov 20 2018;10(11). <https://doi.org10.3390/nu10111801>.

3. Thuesen B, Husemoen L, Fenger M, et al. Determinants of vitamin D status in a general population of Danish adults. *Bone*. Mar 2012;50(3):605-10. <https://doi.org10.1016/j.bone.2011.12.016>.

4. Sayers A, Tilling K, Boucher BJ, Noonan K, Tobias JH. Predicting ambient ultraviolet from routine meteorological data; its potential use as an instrumental variable for vitamin D status in pregnancy in a longitudinal birth cohort in the UK. *Int J Epidemiol*. Dec 2009;38(6):1681-8. <https://doi.org10.1093/ije/dyp237>.

5. Mäkelä J-A, Toppari J. Spermatogenesis. In: Simoni M, Huhtaniemi I, eds. *Endocrinology of the Testis and Male Reproduction*. Cham: Springer International Publishing; 2017:1-39.

**Supplementary Figure 3: Vitamin D levels according to date at sperm ejaculation**

**Vitamin D levels according to date at sperm ejaculation.** Median vitamin D levels according to date of the clinical visit, where the sperm ejaculate was provided and analysed, illustrating the seasonality of vitamin D levels.





**Supplementary Figure 4: Vitamin D levels according to month at sperm ejaculation**

**Vitamin D levels according to month at sperm ejaculation.** Median vitamin D levels (horizontal line) with interquartile range (boxes) according to month of the clinical visit, where the sperm ejaculate was provided and analysed illustrating that calendar month was suitable for predicting vitamin D levels.



| **Supplementary Table S1. Measured vitamin D.** Biomarkers of male fecundity according to measured vitamin D levels at sperm ejaculation in 1047 young men from the fetal programming of semen quality (FEPOS) cohort, nested within The Danish National Birth Cohort, Denmark, 1998 - 2019. | | | | | |  |
| --- | --- | --- | --- | --- | --- | --- |
|  |  |  |  |  |  |  |
|  | Vitamin D levels | | | | |  |
|  | < 25 nmol/l | 25-50 nmol/l | 50-75 nmol/l | > 75 nmol/l | |  |
| n (%) | 149 (14.2) | 427 (40.8) | 365 (34.9) | 106 (10.1) | |  |
| **Semen characteristics** | |  |  |  | |  |
| Volume (ml) | 2.5 (1.9; 3.3) | 2.6 (1.9; 3.5) | 2.9 (1.9; 3.9) | 2.8 (2.1; 3.8) | |  |
| Concentration (mill/ml) | 37.8 (19.4; 63.5) | 36.8 (19.3; 75.3) | 40.8 (16.7; 74.3) | 41.0 (22.9; 63.9) | |  |
| Total sperm count (mill) | 98.5 (39.4; 167.8) | 96.6 (46.2; 196.5) | 110.8 (40.1; 214.0) | 121.6 (55.7; 215.5) | |  |
| Motility (PR %) | 60.6 (50.6; 69.8) | 64.0 (53.9; 74.0) | 65.0 (53.0; 74.0) | 59.5 (51.0; 73.6) | |  |
| Morphology (% normal) | 6.3 (3.6; 11.0) | 7.0 (4.0; 11.0) | 6.0 (2.0; 10.0) | 5.5 (3.6; 9.9) | |  |
| DFI (%) | 9.2 (6.2; 13.0) | 9.0 (6.8; 13.8) | 9.9 (6.3; 13.0) | 10.0 (7.3; 15.1) | |  |
| HDS (%) | 9.0 (7.0; 12.8) | 9.0 (6.0; 13.0) | 9.5 (7.0; 13.0) | 9.0 (7.0; 12.5) | |  |
| **Testes volume** |  |  |  |  | |  |
| Average testes volume (ml) | 15.0 (11.0; 20.0) | 15.0 (12.0; 20.0) | 15.0 (12.0; 20.0) | 15.0 (11.8; 20.0) | |  |
| **Reproductive hormones** |  |  |  |  | |  |
| Testosterone (nmol/L) | 17.8 (14.2; 21.0) | 18.1 (15.0; 22.0) | 18.4 (15.1; 23.0) | 17.8 (14.4; 22.1) | |  |
| Estradiol (pmol/l) | 53.4 (39.3; 73.4) | 53.5 (35.6; 72.8) | 51.6 (32.5; 73.0) | 49.4 (28.8; 77.9) | |  |
| SHBG (nmol/l) | 30.2 (23.0; 39.7) | 32.0 (24.0; 41.0) | 33.7 (27.0; 42.0) | 35.0 (27.9; 43.4) | |  |
| FSH (IU/l) | 3.4 (2.4; 4.6) | 3.5 (2.5; 5.0) | 3.5 (2.5; 5.2) | 4.2 (2.4; 6.0) | |  |
| LH (IU/l) | 4.8 (3.7; 6.3) | 5.1 (4.1; 6.5) | 5.0 (3.9; 6.7) | 5.3 (4.0; 6.8) | |  |
| Free testosterone (nmol/L) | 0.4 (0.3; 0.5) | 0.4 (0.3; 0.5) | 0.4 (0.3; 0.5) | 0.4 (0.3; 0.5) | |  |
| Values are presented as p50 (IQR). All percentiles are pseudo percentiles calculated from the average of five values | | | | |  |  |
| Abbreviations: p50, 50^th^ pseudo percentile. IQR, pseudo intra quartile range. DFI, DNA fragmentation index. HDS, high DNA stainability. SHBG, Sex-hormone binding globulin. FSH, Follicle-stimulating hormone. LH, Luteinising hormone. IU, international units. | | | | | |  |

| **Supplementary Table S2. Estimated vitamin D**. Biomarkers of male fecundity according to estimated vitamin D levels at initiation of spermatogenesis in 1047 young men from the fetal programming of semen quality (FEPOS) cohort, nested within The Danish National Birth Cohort, Denmark, 1998 - 2019. | | | | | |  |
| --- | --- | --- | --- | --- | --- | --- |
|  |  |  |  |  |  |  |
|  | Vitamin D levels | | | | |  |
|  | < 25 nmol/l | 25-50 nmol/l | 50-75 nmol/l | > 75 nmol/l | |  |
| n (%) | 155 (14.8) | 407 (38.9) | 370 (35.3) | 115 (11.0) | |  |
| **Semen characteristics** | |  |  |  | |  |
| Volume (ml) | 2.5 (1.9; 3.4) | 2.7 (1.9; 3.6) | 2.7 (1.9; 3.8) | 2.7 (2.0; 3.7) | |  |
| Concentration (mill/ml) | 36.0 (18.6; 62.6) | 37.4 (19.0; 75.4) | 40.0 (17.2; 72.7) | 41.6 (24.6; 65.0) | |  |
| Total sperm count (mill) | 90.7 (38.2; 166.5) | 101.8 (47.4; 201.5) | 105.9 (38.9; 210.7) | 119.2 (55.2; 207.6) | |  |
| Motility (PR %) | 61.4 (51.9; 72.2) | 63.8 (52.9; 73.5) | 65.3 (53.3; 74.0) | 59.1 (50.9; 72.7) | |  |
| Morphology (% normal) | 6.7 (3.8; 11.4) | 7.0 (4.0; 11.0) | 5.5 (2.0; 9.0) | 5.7 (3.2; 9.9) | |  |
| DFI (%) | 9.6 (6.0; 13.0) | 9.0 (6.0; 13.0) | 10.0 (7.0; 13.4) | 10.1 (7.8; 14.3) | |  |
| HDS (%) | 9.0 (7.0; 13.0) | 9.0 (6.2; 13.0) | 9.4 (7.0; 13.2) | 9.7 (7.0; 13.3) | |  |
| **Testes volume** |  |  |  |  | |  |
| Average testes volume (ml) | 15.0 (11.2; 20.0) | 15.0 (12.0; 20.0) | 15.0 (12.0; 20.0) | 15.2 (12.0; 20.0) | |  |
| **Reproductive hormones** |  |  |  |  | |  |
| Testosterone (nmol/L) | 17.6 (14.0; 21.0) | 18.2 (15.1; 22.0) | 18.4 (14.9; 23.0) | 17.6 (14.1; 22.4) | |  |
| Estradiol (pmol/l) | 53.7 (40.1; 73.2) | 54.2 (36.2; 74.2) | 51.2 (32.6; 72.0) | 49.2 (26.8; 74.0) | |  |
| SHBG (nmol/l) | 30.0 (23.0; 40.0) | 32.9 (24.0; 41.0) | 33.0 (26.7; 42.0) | 34.4 (28.0; 42.2) | |  |
| FSH (IU/l) | 3.4 (2.4; 4.6) | 3.5 (2.5; 5.1) | 3.5 (2.5; 5.0) | 4.2 (2.4; 5.8) | |  |
| LH (IU/l) | 5.1 (3.7; 6.5) | 5.1 (4.1; 6.5) | 5.0 (3.9; 6.6) | 5.1 (3.9; 6.8) | |  |
| Free testosterone (nmol/L) | 0.4 (0.3; 0.5) | 0.4 (0.3; 0.5) | 0.4 (0.3; 0.5) | 0.4 (0.3; 0.5) | |  |
| Values are presented as p50 (IQR). All percentiles are pseudo percentiles calculated from the average of five values | | | | |  |  |
| Abbreviations: p50, 50^th^ pseudo percentile. IQR, pseudo intra quartile range. DFI, DNA fragmentation index. HDS, high DNA stainability. SHBG, Sex-hormone binding globulin. FSH, Follicle-stimulating hormone. LH, Luteinising hormone. IU, international units. | | | | | |  |
