# Supplementary material for: Vitamin D levels and biomarkers of male fecundity: A study from the Danish National Birth Cohort
Source: Andrology. 2025 May 18;14(3):847–62. doi: 10.1111/andr.70061 (PMC12917574; doi:10.1111/andr.70061)
Supplement: Supplementary file 2 — Supporting Information [file ANDR-14-847-s002.pdf]

BASIC HUMAN SEMEN EXAMINATION – CHECKLIST FOR AUTHORS

When publishing results of basic human semen examination in Andrology, it is requested that it is transparent which laboratory methods were used and that these methods are adequate for the purpose. Thus, the journal requests that methods are clearly described in the manuscript. Additionally, the authors must also fill in the semen examination methodology checklist. The checklist is for ejaculate examination (modified from [1]) and it is based on the ISO Standard on basic semen examination [2], the current WHO recommendations ("WHO6")[3], and on general scientific standards. Essential training for laboratory personnel is described in [4]. For data obtained by Computer Assisted Sperm Analysis (CASA) requirements for validation, verification and personnel training are described in [5].

A deviation from this checklist does not necessarily mean that the study cannot be accepted for publication. Still, deviations must be transparently described in the manuscript, including their impact on the accuracy and measurement uncertainty of the data. This is essential to allow the reader to evaluate the quality of the analyses performed. For studies not reporting all characteristics of a basic semen examination, the checklist includes the option 'Not applicable to the study'.

All manuscripts that report results from basic human semen examination should be accompanied by a signed copy of the checklist at the initial submission. That includes studies that report clinical, experimental, and epidemiological results, as well as manuscripts potentially describing other types of studies.

Any scientific rationale for not complying with the guidelines, which is not included in the Materials and Methods section of the manuscript, must be substantiated to the Editor and Reviewers.

REFERENCES

1. Björndahl, L., et al., *Standards in semen examination: publishing reproducible and reliable data based on high-quality methodology.* Hum Reprod, 2022.
2. International Organization for Standardization, *ISO 23162:2021 Basic semen examination — Specification and test methods*. 2021, Geneva: ISO. 32.
3. World Health Organization, *WHO laboratory manual for the examination and processing of human semen*. 6th ed. 2021, Geneva: World Health Organization.
4. Mortimer, D., *Practical Laboratory Andrology*. 1994, Oxford: Oxford University Press. 393.
5. ESHRE Special Interest Group in Andrology, *Guidelines on the application of CASA technology in the analysis of spermatozoa*, in *Hum Reprod*, S. Mortimer, D. Mortimer, and L. Fraser, Editors. 1998. p. 142-5.

1. PATIENTS		EXPLANATION FOR NON-COMPLIANCE
NOT APPLICABLE TO THE STUDY		
X 1.1	The studied population (e.g., patients or volunteers) has been declared in the manuscript, together with the recruitment method and inclusion and exclusion criteria.	X
X 1.2	In a study concerning couples being investigated for infertility, the following is specified in the manuscript: fertility status of the female partner; and all investigations carried out in the male partner.	N/A
X 1.3	If used in the manuscript, the term 'male factor' is completely defined.	N/A
X 1.4	Reference limits provided in WHO5 or 5th percentile of the distribution of semen examination results in WHO6 have not been used to define a man as fertile or infertile.	X

2. GENERAL ASPECTS		EXPLANATION FOR NON-COMPLIANCE
☐ NOT APPLICABLE TO THE STUDY		
☐ 2.1	Patients were instructed to maintain 2–7 days of sexual abstinence before collecting an ejaculate for investigation.	Comment: The participants were instructed to maintain a minimum of 2-3 days of sexual abstinence. For the entire FEPOS cohort, in total n=366 participants maintained less than 2 days of sexual abstinence; n=340 participants maintained the recommended 2-3 days of sexual abstinence; and n=347 participants maintained more than 3 days of sexual abstinence before collecting the semen sample for investigation. Abstinence time was recorded at the clinical visit and taken into account all analyses.
X 2.2	Patients were informed about the importance of reporting any missed ejaculate fractions, and their responses were noted on the laboratory record.	X

2. GENERAL ASPECTS		EXPLANATION FOR NON-COMPLIANCE
☐ 2.3	Ejaculates were collected at the laboratories.	The samples were collected<br>X At the home of the subject.<br>X At another location:<br>Comment: Participants in FEPOS collected the semen sample either at home (n=138) or at the clinic (n=910). Though semen sample collection at the clinics were encouraged, participants living within an hour drive from the clinic had the opportunity to collect the semen sample at home. All analyses were adjusted for place at semen sample collection (at home or in the clinic).<br><br>Time from ejaculation to analysis in minutes was recorded at the clinical visit regardless of place of semen sample collection. For the entire FEPOS cohort, n=35 semen samples were analysed before 30 min; n=751 samples were analysed between 30-60 min; n=260 semen samples were analysed after 60 min. Time from ejaculation to analysis was considered in analyses investigating total motility.<br><br>For specimens not collected at the laboratory:<br>1. Were patients instructed to avoid cooling (under 20 °C) or heating (above 37 °C) the semen specimen during transport to the laboratory? X Yes; ☐ No<br>1. Were patients instructed to deliver the semen specimen to the laboratory within 60 minutes? X Yes; ☐ No
X 2.4	In the laboratory, specimens were kept at 37 °C before initiation of and during the analysis in case of sperm motility assessment.	X
X 2.5	For specimens collected adjacent to the laboratory, analysis was initiated after completion of liquefaction and within 30 min after ejaculation. If some of the specimens were collected at the laboratory and others collected at home, the influence on the data is declared and discussed in the manuscript.	X
X 2.6	Liquefaction was first checked within 30 min after ejaculation.	X
X 2.7	Volume was assessed by weighing.	X
☐ 2.8	Viscosity was measured using a wide-bore pipette or a glass rod.	Comment: Viscosity was not measured, rather it was visually assessed as normal or abnormal.

2. GENERAL ASPECTS	EXPLANATION FOR NON-COMPLIANCE
☐ 2.9 pH was assessed by spreading a drop of well-mixed semen on a pH test strip (with the range of 6.0-10.0)	N/A
X 2.10 All staff members who performed the analyses have been trained in basic semen analysis (ESHRE Basic Semen Examination Course—or equivalent—with further in-house training to establish competency) and regularly participate in internal quality control.	X
X 2.11 When more than one method exists for a particular assessment, only one was used in the study.	X
X 2.12 For a multicenter study, all laboratories used the same method or variable methods are declared in the manuscript.	X

3. SPERM CONCENTRATION ASSESSMENT	EXPLANATION FOR NON-COMPLIANCE
NOT APPLICABLE TO THE STUDY	
X 3.1 Semen aliquot to be diluted for sperm concentration assessment was taken with a positive displacement pipette (i.e., a 'PCR pipette') using a recommended diluent (state which diluent: ____________________).	X
X 3.2 Only standard dilutions were used (1:100, 1:50, 1:20 or 1:10, i.e. 1+99, 1 + 49, 1 + 19 or 1 + 9).	Comment: Standard dilutions were used where applicable; however, in very few cases 1:1, 1:2, and 1:5 was used.
☐X 3.3 Sperm concentration was assessed using counting chamber of type hemocytometer:<br><br>X 3.3.1 Counting chamber with Improved Neubauer ruling.<br><br>☐ 3.3.2.a Other hemocytometer:<br>____________________________<br><br>☐ 3.3.2.b If other hemocytometers were used, adjusted calculation factors were employed	X
X 3.4 Hemocytometers were allowed to rest for 10–15 min in a humid chamber to enable sedimentation of the suspended spermatozoa onto the counting grid before counting.	X
X 3.5 Sperm counting was done using phase contrast microscope optics (200–400×).	X
X 3.6 Comparisons were made between replicate counts (two dilutions, one count of each), and the two counts were within the acceptable variations	X
X 3.7 At least 200 spermatozoa were counted in each replicate assessment.	X

4. SPERM MOTILITY ASSESSMENT		EXPLANATION FOR NON-COMPLIANCE
☐ NOT APPLICABLE TO THE STUDY		
X 4.1	Motility assessments were performed by laboratory personnel	In case Computer Assisted Sperm Analysis (CASA) was used:<br>• Product name and version of the equipment: ____________<br>• validation and verification procedures ____________<br>• training of personnel: ____________
X 4.2	Motility assessments were performed at 37 °C ± 0.5 °C.	X
X 4.3	Motility assessments were initiated within 30–60 min after sample collection.	Time from ejaculation to analysis in minutes was recorded at the clinical visit regardless of place of semen sample collection. For the entire FEPOS cohort, n=35 semen samples were analysed before 30 min; n=751 samples were analysed between 30-60 min; n=260 semen samples were analysed after 60 min. Time from ejaculation to analysis was taken into account in analyses investigating total motility
X 4.4	Motility assessments were performed using phase contrast microscope optics (200–400×).	X
☐ 4.5	Sperm motility was classified using a four-category scheme: rapid progressive, slow progressive, non-progressive, and immotile.	Comment: The motility was assessed as progressive spermatozoa (a), non-progressive spermatozoa (b) and immotile (c) as recommended in WHO laboratory manual for the Examination and processing of human semen, fifth edition (WHO laboratory manual for the examination and processing of human semen, 2010). The outcome used in the study was progressive (a) motility, which, due to model fit, was assessed as non-progressive spermatozoa (b) + immotile (c) motility.
X 4.6	Motility assessments were done in replicate (two aliquots), and the two were within the acceptable variations	X
X 4.7	The wet preparation was made using<br>a drop of __6___ µl<br>and a coverslip of __18___ × __18___ mm<br>to obtain a preparation depth of __18.5___ µm<br>(must be at least 10 µm depth, but not too deep to allow spermatozoa to move freely in and out of focus; typically ca. 20 µm).	X
X 4.7	At least 200 spermatozoa were assessed in each replicate motility count.	X

4. SPERM MOTILITY ASSESSMENT	EXPLANATION FOR NON-COMPLIANCE
☓ 4.9 At least five microscope fields of view were examined in each replicate count.	X

5. SPERM VITALITY ASSESSMENT	EXPLANATION FOR NON-COMPLIANCE
☓ NOT APPLICABLE TO THE STUDY	
☐ 5.1 A validated supravital stain was used to assess sperm vitality, specify: Klicka eller tryck här för att ange text.	
☐ 5.2 At least 200 spermatozoa were evaluated.	
☐ 5.3 Assessments were done under high magnification (×1000–1250) using a 100× high-resolution oil immersion objective and bright field microscope optics.	

6. SPERM MORPHOLOGY ASSESSMENT	EXPLANATION FOR NON-COMPLIANCE
☐ NOT APPLICABLE TO THE STUDY	Comment: The Centre of Reproductive Medicine in Malmö, Sweden, where the morphology assessments were done, not only participates in external quality control; it acts as a reference laboratory for ESHRE-NAFA (Nordic Association of Andrology) External Quality Control.
☐ 6.1 Tygerberg Strict Criteria were used to evaluate human sperm morphology.	Another classification could be used for scientific studies with specific aims if the classification is described or referenced. Depending on the objective of the study, the evaluation of particular abnormal forms might be useful:
☓ 6.2 Abnormalities are recorded for the four defined regions of the spermatozoon (head, neck/midpiece, tail, and cytoplasmic residue).	X
☓ 6.3 The Papanicolaou staining method adapted for assessing human sperm morphology was used. Other staining methods could be used for specific aims but must then be declared and explained.	X
☓ 6.4 At least 200 spermatozoa were assessed in each ejaculate.	X
☓ 6.5 Assessments were done under high magnification (×1000–1250) using a 100× high-resolution oil immersion objective and bright field microscope optics.	X

7. EXTERNAL QUALITY ASSESSMENT (EQA)		EXPLANATION FOR NON-COMPLIANCE
☐ NOT APPLICABLE TO THE STUDY		
X 7.1	The laboratory participated in EQA for the semen examination methods used to obtain data for the study. Our laboratory/ies participated in EQA regarding:<br>☐ Sperm concentration/sperm number<br>☐ Sperm motility.<br>☐ Sperm vitality<br>☐ Sperm morphology	X<br>The EQA from the European Society of Human Reproduction and Embryology (ESHRE; www.eshre.eu) and its Special Interest Group in Andrology (SIG Andrology)
X 7.2	Name of the EQA scheme:	ESHRE SIG Andrology<br>,

8. OTHER FINDINGS		EXPLANATION FOR NON-COMPLIANCE
☐ NOT APPLICABLE TO THE STUDY		
x 8.1	The presence of abnormal clumping (aggregates and agglutinates) was recorded.	x
x 8.2	Abnormal viscosity was recorded.	x

9. ANALYSING DATA		EXPLANATION FOR NON-COMPLIANCE
☐ NOT APPLICABLE TO THE STUDY		
x 9.1	The actual duration of sexual abstinence (in 'hours' or 'days') was recorded for each specimen and included in the data reported in the manuscript.	X
x 9.2	As a minimum in clinical studies, semen volume, sperm concentration, total number of spermatozoa per ejaculate, and abstinence time are given to reflect sperm production and output; only samples identified as having been collected completely were included in the study.	X
x 9.3	Confounding factors have been considered for statistical analysis: e.g., abstinence time and age, to consider secular or geographical variations in sperm concentration or sperm count.	X
☐ 9.4	If appropriate, optional biochemical markers for prostatic, seminal vesicular, and epididymal secretions were analyzed and reported, both as concentration and total amount.	N/A

9. ANALYSING DATA	EXPLANATION FOR NON-COMPLIANCE
☐ 9.5 Signs of active infection/inflammation were noted and considered in the analysis of data in the study (e.g., presence of non-germ line round cells, inflammatory cells, impaired sperm motility, possibly also anti-sperm antibodies, or reduction of secretory contributions).	Comment: Signs of active inflammation was noted; however, the proportion of samples with the presence of inflammatory cells > 1 mill/ml was 0.7% and therefore, too small to consider in the analyses.
X 9.6 If the manuscript also includes results of other types than those originating from basic semen examination (e.g. sperm DNA damage, acrosome reaction, etc), the methodology used to obtain the results is clearly reported.	X

DECLARATION BY THE CORRESPONDING AUTHOR:

The information provided in this checklist is solemnly declared to be true.

Date: 05-02-2025

Signature: ______________________________

Name: Anne Gaml-Sørensen

Affiliation: Aarhus University, Denmark
